# Whitening toothpaste-induced oral mucosal hypersensitivity reaction resembling oral lichenoid reaction: a clinically diagnosed case report

**DOI:** 10.3389/froh.2025.1601156

**Published:** 2025-07-21

**Authors:** Fumin Zheng, Yang Liu, Mei Yang, Xixi Yu, Wanchun Wang

**Affiliations:** ^1^ Department of Oral Medicine, Qingdao Stomatological Hospital Affiliated to Qingdao University, Qingdao, Shandong, China; ^2^ School of Stomatology, Qingdao University, Qingdao, Shandong, China; ^3^ School of Stomatology, Binzhou Medical University, Yantai, Shandong, China

**Keywords:** oral lichenoid reaction, oral lichen planus, whitening toothpaste, hypersensitive reaction, hypersensitive components

## Abstract

This case report describes an instance of oral mucosal hypersensitive reaction resembling oral lichenoid reaction (OLR) induced by the use of a whitening toothpaste in a 28-year-old female patient presenting with extensive white hypersensitive reaction on the oral mucosa. The clinical features resembled an oral mucosal hypersensitivity reaction with lichenoid morphology. A detailed history revealed a clear temporal association between the onset of the reaction and the use of whitening toothpaste. Symptoms rapidly improved following discontinuation of the product, and a positive rechallenge test further confirmed the causal relationship. In conjunction with a review of the literature, the potential pathogenic mechanisms of abrasive agents (e.g., calcium carbonate), chemical agents [e.g., hydrogen peroxide (HP), sodium lauryl sulfate (SLS)], and other additives in whitening toothpaste are discussed. This case documents the first confirmed association between whitening toothpaste and an oral mucosal hypersensitivity reaction with lichenoid morphology, highlighting the importance of differentiating hypersensitivity mechanisms in lichenoid-like presentations.

## 1 Introduction

Oral mucosal hypersensitivity reactions represent a heterogeneous group of immune-mediated inflammatory disorders (1). Some may clinically mimic oral lichen planus (OLP) or oral lichenoid reactions (OLRs) (2). These reaction are characterized by reticular white striations or keratotic papules but differ etiologically from OLP (3), often triggered by amalgam restorations, pharmacological agents (e.g., antibiotics, antihypertensives, antivirals) (4, 5), or systemic conditions (e.g., hepatic diseases, graft-versus-host disease) (6). While OLRs may arise from both delayed-type hypersensitivity and direct toxic effects, the term “lichenoid morphology” should be reserved for describing histopathological features (e.g., basal cell degeneration, lymphocytic infiltrate) rather than implying a specific pathogenic mechanism (7). Diagnostic confirmation typically relies on elimination strategies: replacement of dental restorations or withdrawal of suspected irritants followed by clinical remission monitoring, as lesion regression post-removal strongly supports an irritant-induced etiology (8, 9). In contrast, OLP constitutes an idiopathic disorder, frequently occurring independently or in conjunction with cutaneous lichen planus. This condition manifests as persistent mucosal reaction refractory to conventional interventions, necessitating long-term immunomodulatory management (10). Notably, OLP demonstrates a predilection for middle-aged females, with reported prevalence rates ranging from 0.1% to 4.0%, and carries potential malignant transformation risks in chronic erosive forms (11). Moreover, it should be noted that OLP generally cannot be completely cured and is highly prone to recurrence, thus demanding long-term and meticulous management to control the symptoms and improve the quality of life for patients (12).

Toothpaste, a ubiquitous oral hygiene product utilized across all age demographics, remains a cornerstone in daily oral care for maintaining gingival health and preventing dental caries. Despite its widespread acceptance, growing evidence has elucidated its potential role in triggering localized mucosal hypersensitivity reactions (13, 14). Although erythema, edema, and ulceration—the hallmark features of hypersensitive contact stomatitis—are well-established manifestations of dentifrice allergens (15), isolated oral mucosal hypersensitivity reactions resembling OLRs remain a rare but distinct clinical entity (16). In existing literature, reports of hypersensitive reactions directly caused by toothpaste in healthy individuals are rare. Current studies, including those by Schlosser and Kroona et al., predominantly focus on assessing the sensitizing potential of toothpastes in patients with pre-existing mucosal conditions such as OLP or OLR (13, 17, 18). These investigations indicate that toothpastes containing mint or cinnamon flavorings may exacerbate clinical manifestations in such patients, as supported by analyses of ingredient-related health considerations (19). Comprehensive literature reviews, however, reveal no documented cases of whitening toothpaste-induced oral mucosal hypersensitivity reactions emerging in individuals without prior mucosal pathology. Herein, we present the first reported case of a hypersensitivity reaction with lichenoid morphology triggered by a commercially available whitening toothpaste in a patient without pre-existing oral disease, emphasizing the need for heightened awareness of oral care products as potential immunogenic triggers. This case further expands the spectrum of oral mucosal hypersensitivity by linking it to whitening toothpaste components, underscoring the importance of allergen identification in managing lichenoid-like presentations.

## 2 Case report

**Figure 1:**
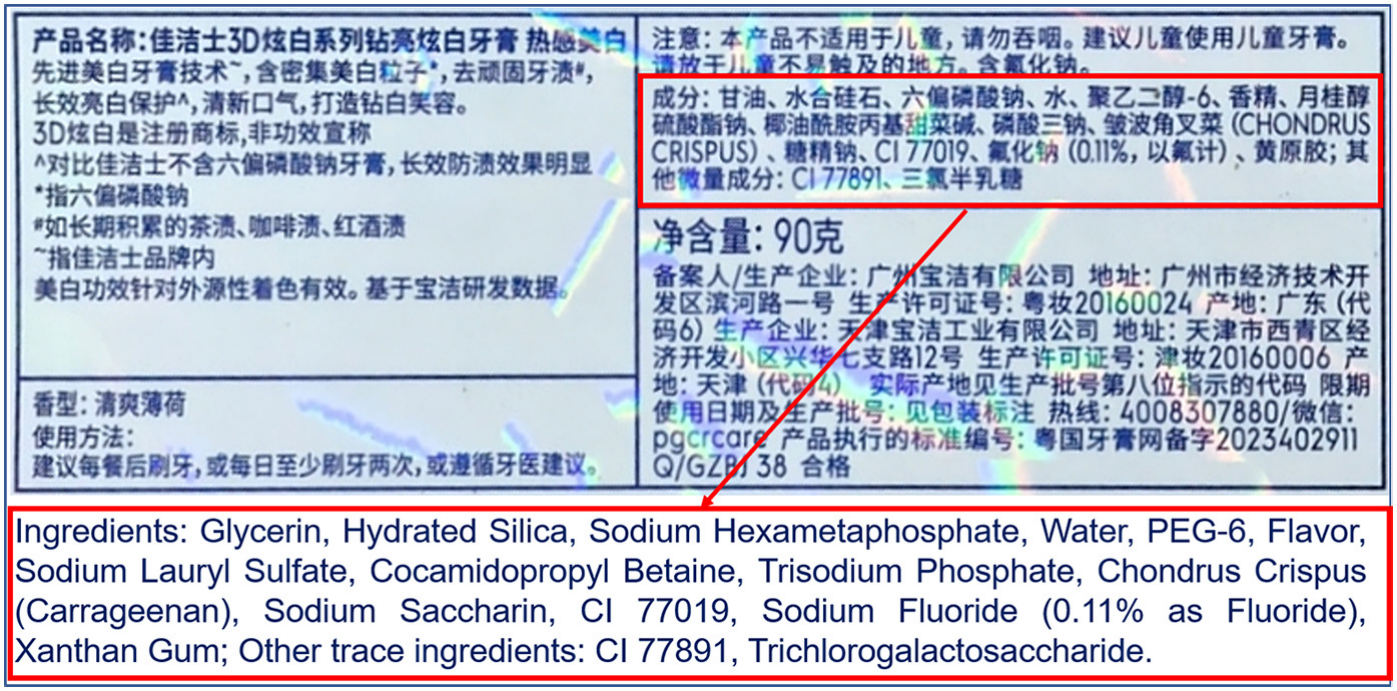
Ingredient label of crest 3D white sparkling whitening toothpaste (procter & gamble, USA). This figure displays the ingredient label of Crest 3D white sparkling whitening toothpaste, highlighting both Chinese and English descriptions. The red rectangular annotations emphasize the active whitening components (e.g., hydrated silica, sodium hexametaphosphate) and fluoride content (sodium fluoride at 0.11% as fluoride), critical for anti-caries efficacy. Additional ingredients include flavoring agents (e.g., cetylpyridinium chloride, saccharin), humectants (glycerin), and stabilizers (xanthan gum). The label also specifies warnings against child ingestion and usage restrictions.

**Figure 2:**
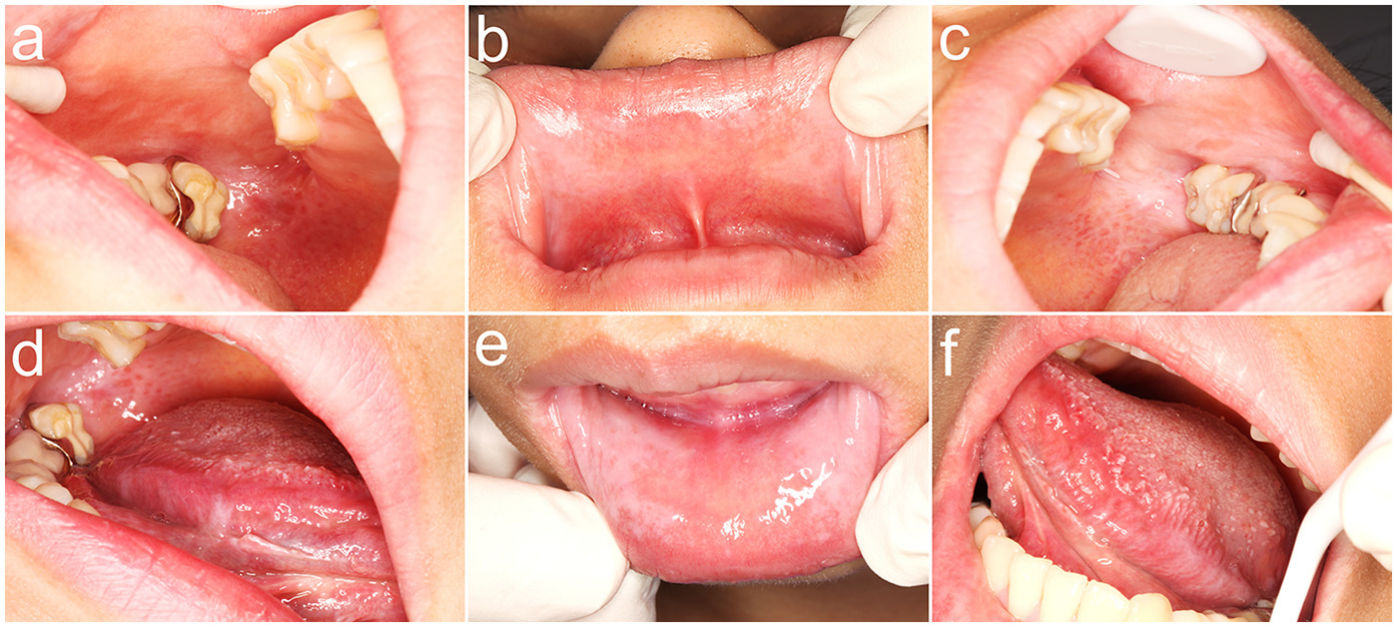
Initial clinical presentation of oral mucosal reaction at diagnosis. **(a)** Diffuse, whitish, cloudy plaques with subtle erythema on the posterior right buccal mucosa; **(b)** Similar cloudy whitish lesions with mild erythema observed on the upper labial mucosa; **(c)** Cloudy white lesion on the left posterior buccal mucosa and reticular white patterns on the soft palate.; **(d)** Linear, branching whitish lesions characteristic of lichenoid morphology on the mid-to-posterior ventral surface of the right tongue; **(e)** Bilateral cloudy whitish lesions with perilesional erythema on the lower labial mucosa; **(f)** Discrete, non-keratotic whitish plaques localized to the posterior ventral surface of the left tongue.

**Figure 3:**
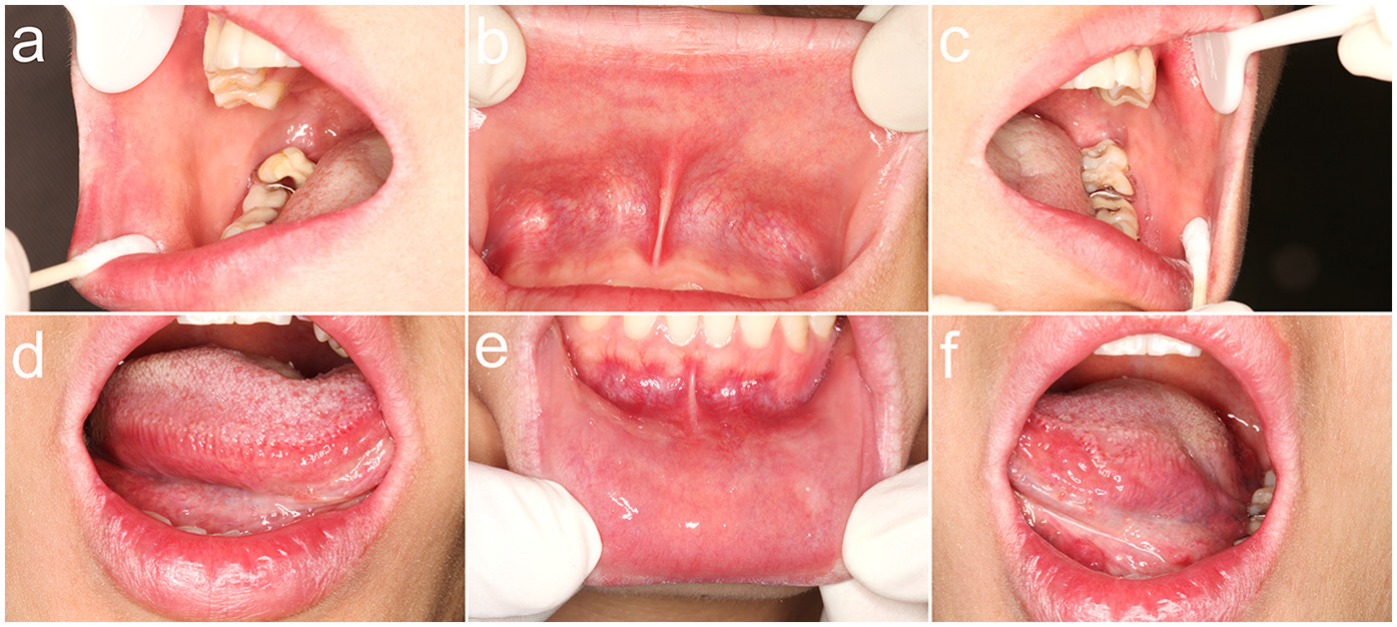
Clinical resolution of oral lesions following 2 weeks of intervention. **(a)** Complete regression of the whitish confluent plaque on the posterior right buccal mucosa, with no residual erythema; **(b)** Absence of the whitish confluent plaque on the upper labial mucosa, without residual erythema; **(c)** Full remission of the whitish confluent plaque on the posterior left buccal mucosa and resolution of the reticulated striae on the soft palate; **(d)** Clearance of the linear, branching whitish lesion on the mid-to-posterior ventral tongue, demonstrating mucosal healing; **(e)** Regression of the whitish confluent plaque on the lower labial mucosa, with no inflammatory sequelae; **(f)** Absence of the whitish confluent plaque on the posterior ventral surface of the left tongue, indicating complete epithelial restitution.

A 28-year-old female patient presented with a primary complaint of whitish discoloration of the oral mucosa persisting for more than one week. The patient reported noticing whitish changes in her oral mucosa approximately one week earlier, without any identifiable predisposing factors. The patient experienced sensitivity when consuming spicy or irritating foods. Notably, the patient had switched to using a whitening toothpaste (Figure 1) just over a week before the onset of symptoms. She denied any history of allergic conditions. Extensive cloudy whitish lesions were observed bilaterally on the buccal mucosa, inner surfaces of the upper and lower lips, and the ventral surfaces of the tongue (Figure 2). The soft palate exhibited scattered reticulated white patterns that were faint in color, non-elevated above the mucosal surface, well-demarcated, smooth, and initially soft in texture, showing no significant difference compared to normal mucosa. No obvious erythema or erosive areas were noted, and Nikolsky's sign was negative, findings that exclude intraepidermal blistering disorders (e.g., pemphigus vulgaris, pemphigus foliaceus) characterized by acantholysis and positive Nikolsky's sign. Palpation of the oral and maxillofacial region revealed no significant lymphadenopathy, reducing the likelihood of systemic conditions such as toxic epidermal necrolysis associated with widespread erythema and systemic toxicity or autoimmune diseases (e.g., systemic lupus erythematosus, graft-versus-host disease) presenting with generalized lymphadenopathy. Hematological and serological examinations were performed to exclude systemic conditions that may mimic lichenoid lesions, such as systemic lupus erythematosus and hepatitis C. The patient denied any disease or local and systemic medication use. A biopsy was not performed due to patient refusal, limiting histopathological confirmation of lichenoid features or *T*-cell infiltration. The provisional diagnosis suggested the possibility of a toothpaste-induced oral mucosal hypersensitivity reaction with lichenoid morphology, distinguishing it from idiopathic OLP and requiring allergen elimination trials for confirmation. The patient received oral loratadine (10 mg once daily for seven consecutive days) and topical dexamethasone sodium chloride rinses (1:50 dilution, 10 ml thrice daily, retained in the oral cavity for 2 min per application), with advice to use pediatric toothpaste (twice daily), typically formulated with fewer irritants and allergens. Following the prescribed treatment and switching to a pediatric toothpaste, the patient reported complete resolution of intraoral sensitivity and discomfort within four days. At the two-week follow-up, the original whitish reaction had completely disappeared (Figure 3). Two months later, the patient self-reinitiated use of the identical toothpaste for three consecutive days, resulting in recurrent lichenoid reaction on the lip mucosa with clinically identical manifestations to the initial episode. Spontaneous resolution occurred within seven days after discontinuation without intervention. The patient was followed up for six months, during which no recurrence was observed. However, longer-term monitoring is recommended to rule out delayed manifestations or cumulative effects of transient exposures. Notably, the patient had bilateral metallic dental prostheses in the oral cavity. These prostheses are known to occasionally induce localized mucosal reactions due to metal ion release or galvanic currents, but the temporal correlation between toothpaste use and lesion onset, along with recurrence upon re-exposure, strongly implicates the whitening toothpaste as the primary etiological factor. The temporal relationship between whitening toothpaste exposure, symptom progression, treatment response, and recurrence upon rechallenge is summarized in Table 1.

**Table 1:** Chronological table of exposure, symptoms, treatment, and outcomes.

Time Point	Event	Details	Remarks
Day 0	Exposure initiation	Patient started using a whitening toothpaste (containing hydrated silica, sodium lauryl sulfate and flavoring agents).	No prior history of oral lesions or allergies.
Day 7	Symptom onset	Whitish discoloration on buccal mucosa, lips, and tongue; sensitivity to spicy foods.	Rapid onset aligns with chemical irritation or hypersensitivity reaction.
Day 8–14	Initial management	Prescribed loratadine (10 mg/day), dexamethasone rinses, and switched to pediatric toothpaste.	Symptoms resolved within 4 days; lesions disappeared by 2-week follow-up.
Week 2	Resolution	Complete disappearance of white lesions and sensitivity.	No histopathological confirmation.
Month 2	Re-exposure test	Patient self-reinitiated original toothpaste for 3 days.	Recurrence of identical lichenoid lesions on lip mucosa confirmed causality.
Week 3	Spontaneous resolution	Lesions resolved within 7 days after discontinuation without intervention.	Supports transient, reversible reaction; no long-term sequelae observed.
Month 6	Final follow-up	No recurrence reported.	Limited follow-up duration; further monitoring recommended.

## 3 Discussion

### 3.1 Diagnostic approach and rationale for non-invasive confirmation

To our knowledge, based on a comprehensive search of PubMed, Embase, and Scopus (from inception to date), no prior cases of oral mucosal hypersensitivity reactions with lichenoid morphology associated with whitening toothpaste have been reported. The critical role of patient history in this diagnosis cannot be overstated. However, our decision to forgo biopsy during initial diagnosis was guided by two critical considerations. First, while histopathological examination remains the gold standard, its invasive nature necessitates careful risk-benefit analysis, particularly when non-invasive diagnostic pathways yield conclusive evidence. Second, we adhered to a stepwise approach advocated in clinical guidelines, prioritizing elimination of potential etiological factors before resorting to irreversible interventions (20). The complete resolution of lichenoid reaction following toothpaste discontinuation, coupled with recurrence upon re-exposure, fulfilled standardized diagnostic criteria for allergen-induced oral lichenoid reactions. This outcome validates the efficacy of our conservative strategy in this specific context (21). This “trigger-retrigger” phenomenon provides robust evidence for a direct etiological relationship, guiding clinicians to scrutinize toothpaste components during differential diagnosis (22, 23).

Groot (2007) highlighted the lack of consensus on optimal patch testing protocols for toothpastes, emphasizing the need to balance dilution concentrations to avoid false results from irritants like abrasives or detergents (15). The limitations of patch testing for oral lesions include histological and functional differences between skin and oral mucosa, pH and permeability variations, distinct immune cell distributions, and risks of false positives due to hapten concentration discrepancies and mucosal absorption, as discussed in Lugović-Mihić et al. (24). In this case, the decision to omit patch testing was justified by the robust clinical trigger-retrigger phenomenon: self-initiated re-exposure to the whitening toothpaste induced recurrent lichenoid reaction with identical features, followed by spontaneous resolution upon discontinuation—a diagnostic framework aligning with non-invasive causal validation criteria. While ingredient analysis remains critical for identifying specific allergens (e.g., SLS, fragrances), the clinical course provided sufficient evidence to confirm the diagnosis without invasive testing. Future studies should prioritize patch testing individual components to refine allergen-specific diagnostics and avoidance strategies (25).

### 3.2 Mechanistic insights into whitening toothpaste-induced oral mucosal hypersensitivity reactions with lichenoid morphology

Whitening toothpaste contains multiple ingredients with potential allergenic or irritant properties, contributing to oral mucosal pathology through distinct mechanisms compared to conventional toothpaste (26). This case highlights the interplay between these components and clinical manifestations in a patient with oral mucosal hypersensitivity reactions with lichenoid morphology.

Calcium carbonate, hydrated silica, and activated charcoal are primary abrasives in whitening formulations, mechanically removing surface stains through friction (27). However, excessive particle size or hardness may compromise the oral mucosal barrier during brushing, leading to microtrauma and subepithelial tissue exposure—a mechanism linked to allergen penetration and immune activation (28). For instance, Jamwal et al. demonstrated that larger abrasive particles correlate with increased enamel roughness (29), while Vaz et al. highlighted their potential to induce mucosal desquamation in hypersensitive individuals (30). Clinically, this underscores the need for particle size standardization in formulations targeting vulnerable populations.

Sodium hexametaphosphate (SHMP) and sodium fluoride (NaF) are widely used in oral care products for their anti-staining and caries-preventive properties, respectively. However, their potential to induce oral mucosal irritation remains a concern. SHMP exhibits dose-dependent mucosal irritation, with high-concentration exposure linked to transient inflammatory responses in nasal and respiratory mucosa. While its application in toothpaste is generally considered safe, prolonged contact or elevated concentrations may disrupt oral mucosal integrity, as demonstrated by Lanigan et al. in preclinical models requiring formal mucosal irritation testing for formulations containing this compound (31). In contrast, NaF demonstrates lower irritancy potential under standard conditions, although Jeng et al. observed cytotoxic effects on human oral mucosal fibroblasts at elevated concentrations (32). This suggests that while sodium fluoride is generally well-tolerated in typical applications, its safety profile may vary depending on exposure levels and cellular contexts.

Hydrogen peroxide (HP), a key oxidative agent, degrades chromophores via free radical generation, achieving whitening through enamel matrix oxidation (33). However, its cytotoxicity disrupts mucosal cell membranes and mitochondria, triggering cytoplasmic leakage and inflammatory mediator release. Marto et al. demonstrated HP's dose-dependent cytotoxicity *in vitro* (34). Chronic exposure exacerbates fibroblasts apoptosis and immune infiltration, processes implicated in OLR pathogenesis. Sardaro et al. further linked high-concentration peroxide gels to accelerated hypersensitivity reactions progression in patients (35). Their findings highlight that HP-induced ROS amplify oxidative stress, compromising mucosal epithelial integrity and promotes inflammatory cascades. These findings advocate for concentration limits in over-the-counter products.

Sodium lauryl sulfate (SLS), a common foaming agent, disrupts epithelial tight junctions by solubilizing membrane lipids, increasing mucosal permeability to allergens (36). This effect is compounded by SLS-induced histamine release from mast cells, amplifying local inflammation. Neppelberg et al. reported that clinically relevant concentrations of SLS *in vitro* induce dual responses in reconstituted human oral mucosa, with low concentrations promoting protective adaptations like epithelial thickening, proliferation, and E-cadherin upregulation, while higher concentrations predominantly cause destructive lesions and structural disruption (37). Clinically, Birant et al. recommended avoiding SLS in patients with pre-existing oral mucosal disorders due to its synergistic role in immune activation (38).

Flavoring agents (e.g., cinnamaldehyde, L-carvone, Lemon) (39, 40), sweeteners (e.g., sodium saccharin), and preservatives (e.g., parabens) act as haptens (41), binding to proteins and activating dendritic cells via toll-like receptors. This initiates Th1/Th17-polarized immune responses, manifesting as vasodilation, edema, and lymphocytic infiltration (42, 43). Chronic exposure drives *T*-cell-mediated lichenoid reactions, a hallmark of OLR. Kroona et al. identified l-carvone as the most prevalent allergen in patch-tested patients with toothpaste-induced hypersensitivity reactions (17), while Bastos et al. linked artificially colored sweets to delayed-type hypersensitivity (44). These data emphasize the need for allergen screening in at-risk users.

### 3.3 Clinical implications and management strategies

The acute sensitivity associated with this reaction temporarily affected the patient's dietary intake and caused discomfort during oral hygiene routines. However, no long-term sequelae were observed following discontinuation of the whitening toothpaste, highlighting the transient nature of the reaction and its minimal impact on overall quality of life. This case underscores the need for heightened clinical vigilance regarding the hypersensitive potential of oral care products. Patients with hypersensitivity tendencies should be advised to use low-irritant toothpaste formulations (e.g., SLS-free, fluoride-reduced) (45). Additionally, clinicians should consider toothpaste-induced oral mucosal hypersensitivity reactions with lichenoid morphology in differential diagnoses for patients presenting with lichenoid mucosal reaction and a history of product changes. Enhanced interdisciplinary collaboration between dermatologists, oral pathologists, and dental researchers is essential to advance understanding of mucosal immunopathology and improve patient outcomes.

## 4 Conclusion

This case report establishes the first documented association between whitening toothpaste and an oral mucosal hypersensitivity reaction with lichenoid morphology, demonstrating its potential as an immunogenic trigger through mucosal barrier disruption and inflammatory activation. The temporal correlation between symptom resolution post-discontinuation and recurrence following rechallenge provides strong evidence for causality, aligning with established diagnostic criteria for hypersensitive/contact reactions. Key components such as abrasives, HP, and SLS likely contribute to epithelial damage and immune sensitization, as supported by their documented mechanisms in prior studies. These findings underscore the importance of clinician awareness in identifying oral care product-related oral mucosal pathologies and advocating for hypoallergenic alternatives in susceptible individuals. Future research should prioritize longitudinal monitoring of similar cases and controlled trials to validate the role of specific toothpaste ingredients in eliciting hypersensitivity with lichenoid morphology.

## Data Availability

The original contributions presented in the study are included in the article/Supplementary Material, further inquiries can be directed to the corresponding authors.
